# Sunset for Leaded Aviation Gasoline?

**DOI:** 10.1289/ehp.121-a54

**Published:** 2013-02-01

**Authors:** Rebecca Kessler

**Affiliations:** **Rebecca Kessler** is a science and environmental journalist based in Providence, RI.

Flying in a small piston-engine plane bears little resemblance to flying in a commercial jet. There are no lines at the airport, no baggage checks, and often less legroom once you duck inside the cabin. You can feel every bump in the tarmac as the plane heads down the runway, every rumble of the engine. Once you are aloft there’s no denying the improbability of flight; the low cruising altitude offers detailed views of the land passing below. The experience is more like traveling in an airborne car than in the insulated cocoon of an airliner. For passengers like this writer, it’s also much more fun.

There’s another less obvious difference. Unlike commercial jets, which use kerosene-based jet fuel, piston-engine aircraft still mostly run on leaded aviation gasoline, or avgas. In fact, avgas is one of the few fuels in the United States that still contain lead,^1^ leaving it the single largest source of lead emissions in the country, according to the U.S. Environmental Protection Agency (EPA).^2^^,^^3^ Concern over the health effects of lead has sparked a contentious effort to finally get the lead out of avgas—something the aviation and petroleum industries have been attempting for more than two decades, to no avail.

## Progress in Reducing Exposure

Lead is a well-documented neurotoxicant that is particularly harmful to children, who are typically exposed when they ingest or inhale lead-containing dust in the home. In recent years, serious harm to cognitive and behavioral functions including intelligence, attention, and motor skills has been demonstrated in children with much less lead in their blood than previously thought to cause harm,^4^^,^^5^ and it is now understood there is no safe level of lead exposure.^6^^,^^7^ In spring 2012 the Centers for Disease Control and Prevention adopted a blood lead “reference value” of 5 µg/dL, reducing by half the level at which intervention is recommended.^8^ An estimated 450,000 U.S. children have blood lead levels above that reference value.^8^

Still, a great deal of progress has been made in reducing lead exposure. Today, the mean blood lead level among U.S. children is 1.2 µg/dL, down from 15 µg/dL in 1976–1980.^9^^,^^10^ This striking reduction tracked with reductions in lead emissions. Lead was banned from residential housing paint in 1978, from plumbing in 1986, and from solder in food cans in 1995. By 1996 it had been phased out of automobile gasoline, which reduced atmospheric lead emissions from 1970 levels by 98%. Tightened controls on metal-processing facilities have since reduced emissions further still.^11^^,^^12^

But unlike automobile gasoline, lead in avgas has remained unreg-ulated.^3^ Tetraethyl lead (TEL) is added to avgas to increase octane and thereby prevent “knock,” or uncontrolled fuel detonation, which can damage aircraft engines during flight, compromising safety. Many aircraft engines have been designed to deliver a lot of power while weighing as little as possible, and they rely on high-octane fuels to do so. Today the most widely available avgas is 100-octane low lead, or 100LL, which would be equivalent to 105-octane automobile gasoline (gas sold at the corner station ranges from 87 to 93 octane).^13^ 100LL contains a maximum TEL content of 2.0 g/gal—less than half that of the 115-octane aviation fuel common in the decades following World War II.^2^

“Lead is like the magic elixir for detonation prevention. There’s really no other chemical that precludes detonation like lead does,” says Peter White, head of the new Fuels Program Office in the Federal Aviation Administration (FAA), which the agency established in fall 2012 to identify an unleaded alternative to 100LL.

There are nearly 20,000 airports across the United States, the majority of them small facilities dedicated to so-called general aviation—civilian, noncommercial flight with private or corporate planes, flying schools, sightseeing tours, and the like. The general-aviation fleet includes a diverse menagerie of aircraft built over many decades, from airplanes to experimental craft.

Seventy-one percent of the fleet, about 159,000 aircraft,^14^ have piston engines and typically use avgas—some 225 million gallons of it in 2011, according to U.S. Energy Information Administration data.^15^ Those numbers have been slipping over the years: In 1981 there were nearly 200,000 piston-engine aircraft, and they burned twice the avgas they do today.^14^ Even so, today piston-engine aircraft are the chief source of lead emissions in the United States, emitting 57% of the 964 tons of lead put into the air in 2008, according to the most recent figures from the National Emissions Inventory.^12^

## Lead Emissions from Airports

Monitoring studies have shown that lead in the air on and surrounding five North American airports exceeds background levels.^16^^,^^17^^,^^18^^,^^19^^,^^20^ However, at only one of those airports—the Santa Monica municipal airport in California—was the monitoring conducted in a way that allows for comparison with the 2008 National Ambient Air Quality Standards (NAAQS) for lead. Locales that exceed those standards must enact emission controls. At Santa Monica, the maximum quarterly average concentration of lead was 0.10 µg/m^3^, two-thirds of the NAAQS limit of 0.15 µg/m^3^. This prompted the EPA to note that airports have the potential to push ambient lead concentrations above the NAAQS, even as the agency now considers whether that standard itself should be lowered.^3^

While a little less than half of lead emissions from piston-engine aircraft linger near airports, the remainder disperses far and wide during flight, according to the EPA.^12^ A 2008 nationwide study by investigators with the National Oceanic and Atmospheric Administration showed that unlike other aerosols, airborne lead particles spike on weekends, probably because of recreational flights.^21^ The potential exists for lead from avgas to contaminate water bodies and enter fish tissue, and low-flying piston-engine crop dusters may deposit it directly onto food crops or livestock.^3^

The EPA considers general-aviation pilots and frequent fliers to have the greatest risk of exposure to lead from avgas. But the general public may also be affected. In 2010 the agency estimated that 16 million people live and 3 million children attend school within a kilometer of lead-emitting airports. This population contains a disproportionate percentage of low-income and minority residents, groups already facing an increased risk of lead exposure.^3^ However, living in an upscale neighborhood is no guarantee of safety: Three-bedroom homes in one neighborhood adjacent to Santa Monica municipal airport regularly fetch upwards of $1 million.

In the only study of its kind, published in 2011, Duke University researchers assessed the relationship between child blood lead levels and residential proximity to airports in six North Carolina counties. They found that children living within 500 m of airports where avgas was used had blood lead levels averaging 4.4% higher than children living more than 2,000 m away. Lead exposure decreased in children living within 500–1,000 m of the airports and declined to background levels further away.^22^

Though small, the average increase was enough to push some children in the study above the CDC reference value of 5 µg/dL, according to Marie Lynn Miranda, the study’s lead author, who is now dean of the School of Natural Resources and Environment at the University of Michigan. And while the airports were not conclusively implicated as the source of the excess lead, Miranda says it’s important to reduce any small sources of lead even in the face of bigger ones like deteriorating lead-based paint, imported products, and some drinking water sources. “While lead in aviation gasoline is not in my top three priorities for childhood lead exposure, it is a source of a known neurotoxicant,” she says. “The more we study lead, the more we understand that even very low levels of exposure cause adverse effects.”

The increase potentially attributable to avgas in Miranda’s study could be much higher than the average in certain subgroups of children, says David Bellinger, a professor of neurology at Harvard Medical School, who studies how neurotoxicants affect brain development. While praising the study, Bellinger says he would like to see further research clarifying how such factors as flight paths and prevailing winds might affect exposure patterns (which Miranda says she is planning).

Bellinger points out that although such low levels of lead exposure may not affect any one child in obvious ways—it’s hard to notice the loss of a few IQ points—the effects can add up for the population as a whole. In April 2012 he published a study calculating the cumulative deficit of IQ points among U.S. children aged 0–5 years that were attributable to a variety of chemical exposures and medical conditions. Lead exposure overall accounted for a nationwide loss of nearly 23 million IQ points, a figure exceeded only by the loss attributable to preterm birth.^23^ “That has some societal impact,” Bellinger says. “It’s prudent to interrupt any exposure pathways that can be interrupted. If we know how to prevent exposure from a certain pathway, it’s probably going to be beneficial to do so.”

## The Push to Get the Lead Out

Pressure has been mounting to do something about the lead in avgas since 2003. That’s when Bluewater Network, a non-profit organization that later merged with the environ-mental group Friends of the Earth (FoE), first wrote to the EPA arguing that leaded avgas endangers public health and welfare. In 2006 FoE petitioned the EPA to either officially declare that it does endanger public health by making a so-called “endangerment finding” that would require regulating it under the Clean Air Act, or else conduct the research necessary to make that determination. In April 2010 the agency issued an Advance Notice of Proposed Rulemaking, which described existing data and planned research and requested comment and further information on the subject, but took no further action.^3^

In March 2012 FoE sued to force the agency to decide whether to make an endangerment finding. Four months later the EPA responded, stating it is conducting monitoring and modeling studies to clarify the effect of lead emissions from airports across the nation and to determine how big a role they play in areas failing to meet the NAAQS.^24^^,^^25^ It expects to issue a decision by the end of 2015.

“We feel like there’s enough evidence already that [leaded avgas] is harmful,” says Marcie Keever, FoE’s legal director. “It doesn’t seem to us and many members of the public who live on the fence line of these airports that there is a question that we need to move toward phasing lead out of avgas.” An EPA spokesperson was unable to make agency scientists or policy staff available for comment.

Meanwhile, in 2011 another environmental group called the Center for Environmental Health (CEH) sued several avgas producers and suppliers under California’s unique Safe Drinking Water and Toxic Enforcement Act (a.k.a. Proposition 65) for failing to warn residents near 25 airports about lead emissions. In response to advance notices from CEH, a group of avgas sup-pliers filed a suit of their own, claiming that federal law preempted the use of Proposition 65 against avgas. If CEH’s suit were to succeed, they claimed, it would effectively shut down the sale of avgas in the state and ground 37,000 general-aviation aircraft. A federal court dismissed the suppliers’ suit, but CEH’s Proposition 65 action remains pending.

**Table 1:** Lead Emissions by Sector, 2008

Sector	Lead Emissions (in tons)
Aircraft	571.49
Industrial Processes	248.06
Electric Generation	59.78
Industrial Boilers	48.38
Waste Disposal	11.46
Commercial/Institutional Fuel Combustion	7.40
Solvent Use	5.20
Residential Fuel Combustion	4.60
Commercial Marine Vessels	2.90
Locomotives	2.28
Agricultural Field Burning	0.97
Gas Stations	0.52
Miscellaneous Nonindustrial (Not Elsewhere Classified)	0.40
Nonroad Diesel Equipment	0.03
Bulk Gasoline Terminals	0.02
Source: U.S. Environmental Protection Agency^12^

The lawsuits and the potential for regulation combined with concerns about rising avgas prices, declining use of piston-engine aircraft that burn the fuel, and the future of TEL supplies, which come from a single manufacturer, convinced the aviation industry that the time had come to find an alternative to 100LL, says Rob Hackman, vice president of regulatory affairs at the Aircraft Owners and Pilots Association (AOPA). AOPA, along with the National Air Transportation Association (NATA) and other aviation and petroleum trade groups joined together in the General Aviation Avgas Coalition. In a 2010 comment submitted to the EPA contending a lack of data to support an endangerment finding, the coalition nonetheless asserted its commitment to “an unleaded future.”

Hackman says the general-aviation industry recognizes that a new type of fuel is necessary and is devoting extensive research to develop it. In fact, it has been trying for more than two decades, with the petroleum industry, to remove lead from avgas. For most of that time they insisted that only a “silver-bullet replacement” fuel would do—one usable by every avgas-burning aircraft and fully compatible with existing fuel supply networks, says Hackman. Those criteria proved to be unattainable.

Dozens of promising blends have been examined in full-scale engine testing, but none could satisfy all the performance requirements of 100LL.^2^ “After twenty years of research, no unleaded formulation has been found that can meet the octane needs of the existing fleet while also maintaining the other necessary safety qualities of an aviation gasoline such as vapor pressure, hot- and cold-starting capabilities, material compatibility, water separation, corrosiveness, storage stability, freeze point, toxicity, and a host of other traits necessary to be a true drop-in,” Hackman says.

## Possible Solutions

The industry now accepts that 100LL’s replacement will require tradeoffs and is searching for the least-disruptive alternative, Hackman says. That won’t happen quickly, though. In 2011 the FAA announced its intent to make an unleaded fuel available by 2018.^26^ It also convened the Unleaded Avgas Transition Aviation Rulemaking Committee to address the issue with representatives from the EPA, industry trade groups including AOPA and NATA, fuel producers, and aircraft manufacturers. (FoE declined an invitation to join when it learned committee proceedings would not be public, Keever says.) In 2012 the committee released a report recommending a process and criteria for identifying and approving an unleaded avgas over 11 years.^2^

The FAA has adopted many of the committee’s recommendations and opened the Fuels Program Office to oversee certification of a new fuel. The agency also currently allocates about $2 million per year for research and development—though that’s a fraction of what the committee requested. The process is open-ended, says the FAA’s White. At a minimum he says the agency expects to certify a version of the current 100LL fuel with the lead removed (resulting in a reduced rating of 91–94 octane), which would satisfy a good chunk of the piston-engine fleet. The FAA also plans to evaluate what modifications would enable high-performance engines to burn a lead-free version of 100LL, albeit with a potential loss in performance.

Another option would be to slowly phase the lead out of avgas while developing any necessary engine modifications in tandem. Yet another would be to find a way to make more than one fuel available at airports that would satisfy the low- and high-performance segments of the fleet separately.

Some observers argue that this latter solution has been right under the industry’s nose all along. The majority of the U.S. piston-engine fleet—around 75% is the number generally accepted by the industry, according to White—does not require high-octane avgas at all. Most of these aircraft can run quite happily on ordinary unleaded automobile gasoline, provided it does not contain ethanol. This fuel, often called autogas or mogas, also has the advantages of being cheap and produced in vast quantities, compared with 100LL avgas.

Although most older piston-engine aircraft aren’t factory-certified to run on mogas, owners of some 60,000 aircraft—about one-third of the piston-engine fleet—have already procured the certificates and accompanying fuel-cap stickers that are required for tanking up with mogas, according to Kent Misegades, a North Carolina–based recreational pilot and cofounder of the Aviation Fuel Club, which advocates for access to mogas. Furthermore, he says, nearly all of the latest generation of piston engines come factory-certified to operate on mogas.^27^ As for the remaining quarter or so of piston-engine aircraft that now needs high-octane avgas (the Aviation Fuel Club says these actually account for only 17–20% of the piston-engine fleet), Misegades says most can be modified with existing technology to run on mogas.

## Market Factors

Although gasoline without ethanol is increasingly rare, the biggest barrier to using mogas is that only a handful of U.S. airports offer it, Misegades says. “They want to create some new boutique fuel for these specialized, high-performance engines that are hardly made,” he says of the FAA’s committee. “You could make a big dent in the lead emissions literally within a year by getting more mogas onto the field,” something he says most small European general-aviation airports already do. In October 2012 U.S. congressman Henry Waxman (D–California), the ranking member of the House Energy and Commerce Committee, made a similar case in a letter to the FAA.^28^

White agrees that increasing use of mogas is a good idea, but he says the high-performance aircraft that cannot currently burn it tend to fly more than the less-demanding aircraft that can, and actually consume about 70% of the avgas used, according to unpublished industry estimates. Moreover, the market—not the FAA—dictates what fuels are available, and fuel suppliers believe the market does not support the costs associated with making mogas or any other fuel available alongside 100LL avgas at airports, according to Michael France, director of regulatory affairs at NATA, who served on the FAA committee. The market for avgas is so small, he explains, that most general-aviation airports and their fuel suppliers balk at the cost of adding fuel tanks and other equipment needed to offer a second fuel. “We support a process that identifies the replacement fuel that works best for everyone, and a two-grade solution similar to how the auto industry transitioned is really unworkable in aviation,” France says.

One illustration of the market’s sway is that in 2011 the FAA approved a fuel for use in all piston-engine aircraft with a maximum lead content 19% lower than that of 100LL. The new fuel is called 100VLL, for “very low lead.” White says some 100LL on the market actually meets the new 100VLL standard; in fact, data from 89 samples of 100LL indicate that on average the avgas being sold only slightly exceeds the 100VLL standard. Yet even though lower-lead avgas is already being used by the piston-engine aircraft fleet without complaint, White says he is unaware of any 100VLL being sold separately because there isn’t any specific demand for it. There also is no legal requirement to provide it.

“We can approve fuels, we can certify aircraft and engines to operate on those fuels—we’ve done that before,” says White. “How do we get over the hurdle of getting the market to make that fuel available at the airports? That’s really our big challenge.”

Of course, an endangerment finding by EPA would do just that. Currently, at least one federal agency, the aviation and petroleum industries, and environmental groups all seem to agree that leaded avgas’s days are numbered. What remains to be hashed out is how to go about replacing it, how quickly to proceed, and whether the industry can come up with a solution before the EPA decides whether to regulate. For now, the process appears to be a long-haul flight.
